# Marked augmentation of PLGA nanoparticle-induced metabolically beneficial impact of γ-oryzanol on fuel dyshomeostasis in genetically obese-diabetic *ob/ob* mice

**DOI:** 10.1080/10717544.2017.1279237

**Published:** 2017-02-09

**Authors:** Chisayo Kozuka, Chigusa Shimizu-Okabe, Chitoshi Takayama, Kaku Nakano, Hidetaka Morinaga, Ayano Kinjo, Kotaro Fukuda, Asuka Kamei, Akihito Yasuoka, Takashi Kondo, Keiko Abe, Kensuke Egashira, Hiroaki Masuzaki

**Affiliations:** 1 Division of Endocrinology, Diabetes and Metabolism, Hematology, Rheumatology (Second Department of Internal Medicine), Graduate School of Medicine and; 2 Department of Molecular Anatomy, Graduate School of Medicine, University of the Ryukyus, Okinawa, Japan,; 3 Department of Cardiovascular Medicine and; 4 Department of Cardiovascular Research, Development, and Translational Medicine, Kyushu University Graduate School of Medical Sciences, Fukuoka, Japan,; 5 SENTAN Pharma Inc., Fukuoka, Japan,; 6 Kanagawa Academy of Science and Technology, Kanagawa, Japan, and; 7 Department of Applied Biological Chemistry, Graduate School of Agricultural and Life Sciences, The University of Tokyo, Tokyo, Japan

**Keywords:** Type 2 diabetes mellitus, brown rice, γ-oryzanol, nanotechnology, poly (DL-lactide-co-glycolide), obesity, therapeutic modality

## Abstract

Our previous works demonstrated that brown rice-specific bioactive substance, γ-oryzanol acts as a chaperone, attenuates exaggerated endoplasmic reticulum (ER) stress in brain hypothalamus and pancreatic islets, thereby ameliorating metabolic derangement in high fat diet (HFD)-induced obese diabetic mice. However, extremely low absorption efficiency from intestine of γ-oryzanol is a tough obstacle for the clinical application. Therefore, in this study, to overcome extremely low bioavailability of γ-oryzanol with super-high lipophilicity, we encapsulated γ-oryzanol in polymer poly (DL-lactide-co-glycolide) (PLGA) nanoparticles (Nano-Orz), and evaluated its metabolically beneficial impact in genetically obese-diabetic *ob/ob* mice, the best-known severest diabetic model in mice. To our surprise, Nano-Orz markedly ameliorated fuel metabolism with an unexpected magnitude (∼1000-fold lower dose) compared with regular γ-oryzanol. Furthermore, such a conspicuous impact was achievable by its administration once every 2 weeks. Besides the excellent impact on dysfunction of hypothalamus and pancreatic islets, Nano-Orz markedly decreased ER stress and inflammation in liver and adipose tissue. Collectively, nanotechnology-based developments of functional foods oriented toward γ-oryzanol shed light on the novel approach for the treatment of a variety of metabolic diseases in humans.

## Introduction

Although various types of drugs have been developed, the prevalence of obesity and type 2 diabetes mellitus is increasing worldwide (Moller, 2001; Freedman, 2011; Olokoba et al., 2012). Based on this situation, recent studies emphasize the importance of the healthy dietary habits in prevention and amelioration of obesity-diabetes syndrome (Freedman, 2011; Mozaffarian et al., 2011). Notably, it has been shown that brown rice prevents the onset of type 2 diabetes in humans (Sun et al., 2010; Shimabukuro et al., 2014). Therefore, we focused on the impact of a brown rice-specific bioactive substance γ-oryzanol, an ester mixture of ferulic acid and several kinds of phytosterols (Lerma-Garcia et al., 2009), on therapeutic properties of obesity-diabetes syndrome.

Our previous works demonstrated that γ-oryzanol acts as a chaperone, thereby attenuating the preference for animal fat through suppression of endoplasmic reticulum (ER) stress in hypothalamus (Kozuka et al., 2012). In pancreatic islets from both high fat diet (HFD)-induced and streptozotocin-induced diabetic mice, we also demonstrated that γ-oryzanol ameliorates ER stress and protects β-cells against apoptosis (Kozuka et al., 2015b). ER stress plays an important role in the pathophysiology of obesity-diabetes syndrome in a variety of tissues (Hotamisligil, 2010). In liver, for example, dephosphorylation of translation initiation factor 2α (eIF2α), a critical signaling molecule of ER stress, enhances glucose tolerance via diminishing gluconeogenesis steatosis (Oyadomari et al., 2008). Furthermore, in both liver and adipose tissue, ER stress-related activation of c-Jun N-terminal kinases (JNK) by deletion of an X-box binding protein 1 (XBP1) allele, aggravates insulin receptor signaling, resulting in systemic insulin resistance (Ozcan et al., 2004). Taken together, γ-oryzanol may be a promising tool to suppress exaggerated ER stress for the prevention and treatment of obesity-diabetes syndrome.

Because of its extremely high lipophilicity, γ-oryzanol is hardly absorbed form the intestine (Kozuka et al., 2013). Therefore, in this study, to increase the efficiency of its absorption, we encapsulated γ-oryzanol in polymer poly (DL-lactide-co-glycolide) (PLGA) nanoparticles. PLGA is hydrolyzed in the body, breaking its ester linkages to form lactic acid and glycolic acid monomers that can be easily metabolized by Krebs cycle (Shenderova et al., 1999). Compared to oral suspension of pure drugs, nanoparticle formulation after oral administration markedly augmented maximum plasma concentration as well as area under the plasma concentration-time curve (Sahana et al., 2008). In agreement with this notion, it has been well documented that nanoparticle-mediated drug delivery system using bioabsorbable PLGA nanoparticles markedly enhances therapeutic effects of a variety of compounds in animal models of hindlimb ischemia, pulmonary arterial hypertension, atherosclerosis and acute myocardial infarction (Chen et al., 2011; Ishikita et al., 2016; Koga et al., 2016). However, it is unknown whether the use of oral administration PLGA nanoparticles targeting γ-oryzanol into intestine can be developed as a clinically feasible drug delivery system for obesity-diabetes syndrome. In this context, we assessed the metabolically beneficial impact of γ-oryzanol-encapsulated PLGA nanoparticles (Nano-Orz) on fuel dyshomeostasis in genetically obese-diabetic *ob/ob* mice.

## Methods

### Animals

Five-week-old male *ob/ob* mice obtained from Charles River Laboratories Japan, Inc. (Kanagawa, Japan) were housed at 24 °C under a 12-h/12-h light/dark cycle. The mice were allowed free access to food and water. All animal experiments were approved by the Animal Experiment Ethics Committee of the University of the Ryukyus (No. 5352, 5718 and 5943). Metabolic parameters were measured as previously described (Kozuka et al., 2015b) (Supplemental Methods).

### PLGA nanoparticles

PLGA nanoparticles were prepared using an emulsion solvent diffusion method as previously described (Ishikita et al., 2016) (Supplemental Methods). According to the manufacturer’s instructions (Okada et al., 1991; Kubo et al., 2009), bio-absorption half-life of this product looks approximately 2 weeks in rat tissue. In this study, mean particle size used was 214.8 ± 4.3 nm, and mean zeta potential was −19.4 ± 1.5 mV, respectively. PLGA nanoparticles incorporated with the indocyanine green (ICG), fluorescein isothiocyanate (FITC) or γ-oryzanol were suspended in water and delivered into the stomach by a gavage needle. Fluorescence images were acquired using FMT 4000 In Vivo Imaging System (PerkinElmer, Inc., Waltham, MA) at 1.5, 3, 24, 48, 72 and 96 h after the administration (excitation wavelength: 745 nm/emission wavelength 770–800 nm). We treated *ob*/*ob* mice with FITC-encapsulated PLGA nanoparticles as a negative control. However, this was done in a few subset of experiments because FITC induces inflammation such as contact hypersensitivity depending on CD4 + Th2 cells (Takeshita et al., 2004).

### Administration of γ-oryzanol

Gamma-oryzanol (Wako Pure Chemical Industries, Ltd., Osaka, Japan) was dissolved in 0.5% methyl cellulose solution (Wako Pure Chemical Industries). Gamma-oryzanol (320 μg/g body weight) was delivered into the stomach by a gavage needle every day for 4 weeks.

### Monitoring of preference for dietary fat in mice

Preference for dietary fat was evaluated in the two-foods (chow versus HFD) choice tests as previously described (Kozuka et al., 2012). Briefly, the mice were allowed free access to the chow and the HFD. Intakes of the chow and HFD were measured weekly and analyzed for changes in the preference for dietary fat. HFD preference was calculated according to the following formula: HFD preference = [(HFD intake/total food intake) × 100].

### Fecal and hepatic lipid contents

The feces from each group were collected weekly and dried. Total lipids were extracted from the feces and liver according to the procedure of Folch et al. (Folch & Lebaron, 1956). Triglyceride and total-cholesterol concentrations were measured using the triglyceride E-test kit and total-cholesterol E-test kit, respectively (Wako Pure Chemical Industries).

### IHC analyses and oil red O staining

Immunohistochemistry (IHC) analyses were examined at 4 weeks after starting the treatment as described (Kozuka et al., 2015b) (Supplemental Methods). For Oil red O staining, liver tissues were frozen in optimal cutting temperature (OCT) compounds. The floating liver sections were stained with 0.3% Oil red O solution (Sigma-Aldrich, Traunstein, Germany) and mounted with Fluoromount (Sigma-Aldrich). Then, the slides were processed for hematoxylin counter staining.

### Isolation of pancreatic islets

Pancreatic islets were isolated from mice at 4 weeks after starting the treatment by collagenase digestion (Liberase TL; Roche Diagnostics GmbH, Mannheim, Germany) and purified on a Histopaque gradient (Histopaque 1077; Sigma-Aldrich) as described (Zmuda et al., 2011).

### Quantitative real-time PCR

Gene expression was examined as described (Kozuka et al., 2012) (Supplemental Methods and Supplemental Table 1). All of mRNA samples were collected at 4 weeks after starting the treatment.

### MicroArray analyses

For assays, three representative mice were selected in each group. DNA microarray analysis was performed as previously described (Kamei et al., 2013) (Supplemental Methods). All of the microarray data are Minimum Information About a Microarray Experiment (MIAME) compliant and have been deposited in a MIAME compliant database, namely the National Center for Biotechnology Information (NCBI) Gene Expression Omnibus (GEO) (http://www.ncbi.nlm.nih.gov/geo/, GEO Series accession number GSE86858), as detailed on the MGED Society website (http://www.mged.org/Workgroups/MIAME/miame.html).

### Analysis of composition of gut microbiota

Microbial DNA was extracted from fecal samples using a QIAamp Fast DNA Stool Mini Kit (QIAGEN, Tokyo, Japan), and the V3–V4 region of the 16S rRNA gene was amplified, sequenced and analyzed with the Illumina MiSeq platform (Illumina, San Diego, CA). The amplicon reads were clustered into operational taxonomic units (OTUs) at 97% identity level, and filtered for chimeric sequences using CD-HIT-OTU. The following sequence data analysis was performed as previously described (Satoh et al., 2013).

### Measurement of short-chain fatty acid (SCFA) concentrations in peripheral blood

Short-chain fatty acids (SCFAs) were quantitatively analyzed using a liquid chromatography-tandem mass spectrometry (LC-MS/MS). Plasma sample preparation was performed at 4 weeks after starting the treatment as previously described (Miwa & Yamamoto, 1987). Then, samples were injected into a LC-MS/MS system. Chromatographic separation was achieved using an octadecylsilyl (ODS) column, with two mobile phases, and electrospray ionization in negative ionization with multiplied reaction monitoring (MRM) mode was used.

### Statistical analysis

Data are expressed as the mean ± standard error of the mean (SEM) in independent experiments. One-way analysis of variance (ANOVA) and repeated-measures ANOVA followed by multiple comparison tests (Bonferroni/Dunn method) were used where applicable. Student’s *t*-test was used to analyze the differences between two groups. Differences were considered significant at *p* < 0.05.

## Results

### Tissue distribution of PLGA nanoparticles in mice

To assess the tissue distribution of orally supplied nanoparticles in 8-week-old male mice, ICG-encapsulated PLGA nanoparticles (Nano-ICG) (3 mg/mouse) or ICG (3 mg/mouse) were orally administered. Due to the relative instability of ICG, we observed ICG accumulation for 4 days after administration of Nano-ICG.

**Figure 1.**
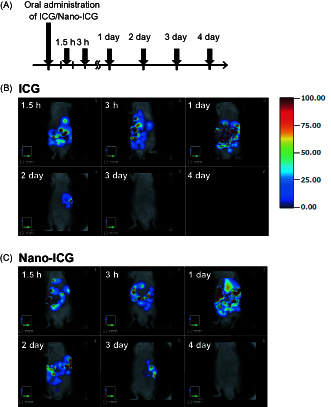
Tissue distribution of PLGA nanoparticles after oral administration in mice. (A) Experimental protocol. C57BL/6J mice were treated orally with ICG or Nano-ICG. Alive mice were non-invasively imaged by FMT at 1.5 h, 3 h, 1, 2, 3 and 4 day after treatment. Tissue distribution of ICG (B) and Nano-ICG (C) were evaluated for 4 days after oral administration. The color scale bar is represented in arbitrary unit from blue (low intensity of fluorescence signal) to red (high intensity of fluorescence signal).

ICG showed peak signal at 1 day and decreased rapidly within 2 days. Nano-ICG also showed peak signal at 1 day, but it continued to distribute longer than ICG (Figure 1). As previously shown (Semete et al., 2010; Chereddy et al., 2016; Navarro et al., 2016), Nano-ICG was distributed mainly in the liver followed by intestine, kidney and spleen (Figure 1).

### Effects of γ-oryzanol-encapsulated PLGA nanoparticles (Nano-Orz) on body weight in mice

**Figure 2.**
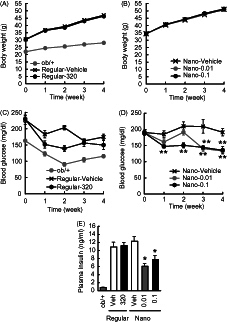
Effects of Nano-Orz on body weight and blood glucose level in *ob*/*ob* mice. Effects of regular γ-oryzanol (320 μg/g body weight/day) (A) and Nano-Orz (0.01 and 0.1 μg/g body weight/day) (B) on body weight in *ob*/*ob* mice. Effects of regular γ-oryzanol (C) and Nano-Orz (D) on blood glucose levels in *ob*/*ob* mice in the fed state. (E) Effects of Nano-Orz (0.01 and 0.1 μg/g body weight/day) on plasma insulin levels. Data are expressed as mean ± SEM (*n* = 8). *p < 0.05, **p < 0.01 compared with control mice. ANOVA followed by multiple comparison tests (Bonferroni/Dunn method) was used.

Five-week-old male *ob/ob* mice were treated with regular γ-oryzanol or Nano-Orz for 4 weeks, and their body weight, food intake and blood glucose level in fed states were monitored. Neither regular γ-oryzanol nor Nano-Orz affected body weight gain in *ob/ob* mice (Figures 2A and B). There were no differences in daily food intake between mice administered regular γ-oryzanol and Nano-Orz (Regular-Veh, 38.4 ± 3.4 g/week; Regular-Orz, 38.4 ± 3.1 g/week; Nano-Veh, 36.1 ± 3.0 g/week; Nano-Orz 0.01, 36.6 ± 3.0 g/week; Nano-Orz 0.1, 36.2 ± 2.7 g/week).

### Effects of Nano-Orz on glucose metabolism in mice

Regular γ-oryzanol showed no effect on blood glucose levels (Figure 2C). On the other hand, in mice treated with Nano-Orz, blood glucose levels were significantly lower than that in mice treated with vehicle (Figure 2D). Regular γ-oryzanol did not affect plasma insulin levels, while Nano-Orz significantly decreased plasma insulin revels (Figure 2E).

**Figure 3.**
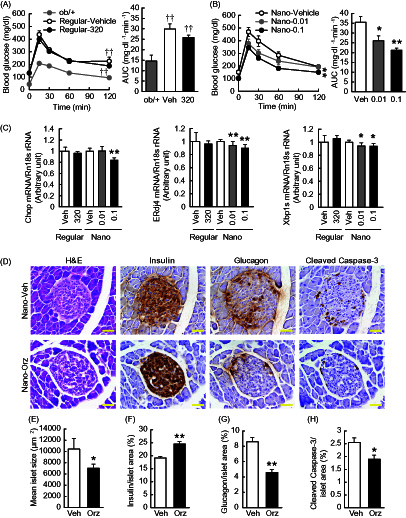
Effects of Nano-Orz on glucose homeostasis in *ob*/*ob* mice. Blood glucose levels and AUC during GTT in mice treated with regular γ-oryzanol (A) and Nano-Orz (B) for 2 weeks. Data are expressed as mean ± SEM (*n* = 8). †† *p* < 0.01 compared with ob/+ mice. *p < 0.05, **p < 0.01 compared with Nano-Vehicle-treated mice. ANOVA and repeated-measures ANOVA followed by multiple comparison tests (Bonferroni/Dunn method) were used. (C) Levels of mRNA in the hypothalamus are shown for Chop, ERdj4 and Xbp1s. The mRNA levels were determined using real-time PCR. Values were normalized to that of 18S rRNA. (D) IHC analyses of isolated pancreatic islets. Serial paraffin-embedded sections were stained with anti-insulin, anti-glucagon and anti-cleaved caspase-3 antibodies. Scale bar, 20 μm; magnification, ×400. (E–H) Mean islet size (E) and the ratios of insulin-positive area (F), glucagon-positive area (G) and cleaved caspase-3-positive area (H) to the total islet area were calculated (*n* = 3; 156–203 islets), respectively. Data are expressed as mean ± SEM. *p < 0.05, **p < 0.01, versus control mice. ANOVA followed by multiple comparison tests (Bonferroni/Dunn method) was used. Student’s *t*-test was used to analyze the differences between two groups.

To further evaluate the effect of Nano-Orz on glucose metabolism, oral glucose tolerance test (OGTT) was performed at 1.5 weeks after starting the treatment. When glucose was delivered into the stomach, there was no significant difference in blood glucose between the mice treated with vehicle and regular γ-oryzanol (Figure 3A). In mice treated with Nano-Orz, blood glucose levels were decreased significantly compared to the vehicle-treated mice (Figure 3B left). A significant reduction was also observed in the area under the curve (AUC) for glucose during the OGTT in mice treated with Nano-Orz (Figure 3B right).

To investigate the effect of Nano-Orz on ER stress in pancreatic islets, we analyzed the mRNA levels of ER stress-responsive genes including ER resident DNAJ 4 (ERdj4) and the spliced form of Xbp1 (Xbp1s). Nano-Orz significantly decreased the mRNA levels of Chop, ERdj4 and Xbp1s in pancreatic islets in *ob/ob* mice (Figure 3C). Nano-Orz-related beneficial effect of islet protection was confirmed by IHC analyses (Figures 3D–H). Positive cells for cleaved caspase-3 (representative marker for apoptosis) were decreased by Nano-Orz (Figures 3D and H). Moreover, enlargement of islet in *ob*/*ob* mice (Bock et al., 2003) was ameliorated by Nano-Orz (Figure 3E). Of note, Nano-Orz augmented the intensity of insulin staining and increased the ratio of insulin-positive area to total islet area (Figures 3D and F), while decreased the ratio of glucagon-positive area to total islet area (Figures 3D and G), indicating the significant improvement in pancreatic islet function.

### Effects of Nano-Orz on insulin sensitivity in mice

**Figure 4.**
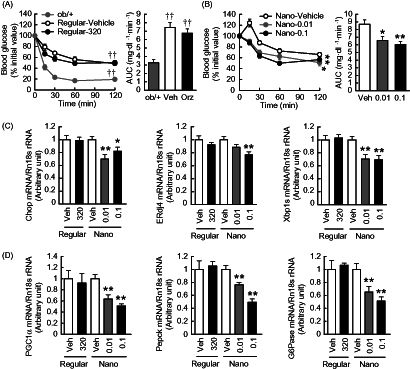
Effects of Nano-Orz on insulin tolerance and hepatic gene expressions related to ER stress and glucose homeostasis in *ob*/*ob* mice. Blood glucose levels and the AUC during ITT treated with regular γ-oryzanol (A) and Nano-Orz (B) for 3 weeks. Data are expressed as mean ± SEM (*n* = 8). †† *p* < 0.01 compared with ob/+ mice. *p < 0.05, **p < 0.01 compared with Nano-Vehicle-treated mice mice. (C, D) Expression levels of mRNA for Chop, ERdj4, Xbp1s (C), PGC1α, Pepck and G6Pase (D) in liver (*n* = 8). The mRNA levels were determined by real-time PCR and normalized by those of 18S rRNA. Data are expressed as mean ± SEM. *p < 0.05, **p < 0.01 versus vehicle-treated mice (Veh). ANOVA and repeated-measures ANOVA followed by multiple comparison tests (Bonferroni/Dunn method) were used.

To assess the insulin sensitivity in mice treated with Nano-Orz, insulin tolerance test (ITT) was performed at 2.5 weeks after starting the treatment. When mice was injected into the intraperitoneal cavity with insulin, blood glucose levels were significantly lower in mice treated with Nano-Orz, whereas there was no change in blood glucose levels in mice treated with regular γ-oryzanol, compared to those in mice treated with vehicle (Figures 4A and B). We also observed a significant reduction in the AUC for glucose during the ITT in mice treated with Nano-Orz (Figure 4B right).

To assess the effect on insulin resistance in liver, we analyzed the mRNA levels of ER stress-responsive genes as well as gluconeogenic genes including peroxisome proliferator activated receptor γ coactivator-1α (PGC-1α), phosphoenolpyruvate carboxykinase (PEPCK) and glucose 6-phosphatase (G6Pase) (Salvado et al., 2015). The mRNAs of ER stress-responsive genes were highly expressed in liver of *ob/ob* mice compared to those of *ob/+ *mice (Chop, 1.6 fold elevated; ERdj4, 1.4 fold elevated; Xbp1s, 1.3 fold elevated versus *ob/+ *mice). Nano-Orz significantly decreased the mRNA levels of Chop, ERdj4 and Xbp1s in liver in *ob/ob* mice, whereas regular γ-oryzanol showed no effect on mRNA levels of these genes (Figure 4C). In parallel, mRNA levels of PGC-1α, PEPCK and G6Pase were significantly decreased by the treatment with Nano-Orz (Figure 4D).

In adipose tissue, HFD-induced ER stress and inflammation play a pivotal role in the development of insulin resistance (Salvado et al., 2015). Therefore, we analyzed the mRNA levels of ER stress-responsive genes and pro-inflammatory genes including tumor necrosis factor-α (TNFα) and interleukin-6 (IL-6), and monocyte chemoattractant protein-1 (MCP-1) and peroxisome proliferator-activated receptor γ (PPARγ), a master regulator of adipocyte differentiation. Both regular γ-oryzanol and Nano-Orz significantly decreased the mRNA levels of Chop, ERdj4, Xbp1s, TNFα, IL-6 and MCP-1 in mesenteric fat in *ob/ob* mice (Supplemental Figures 1A and 1B). Nano-Orz showed a trend to decrease mRNA level of PPARγ, while regular γ-oryzanol showed no effects (Supplemental Figure 1C).

### Effects of Nano-Orz on lipid metabolism in mice

**Figure 5.**
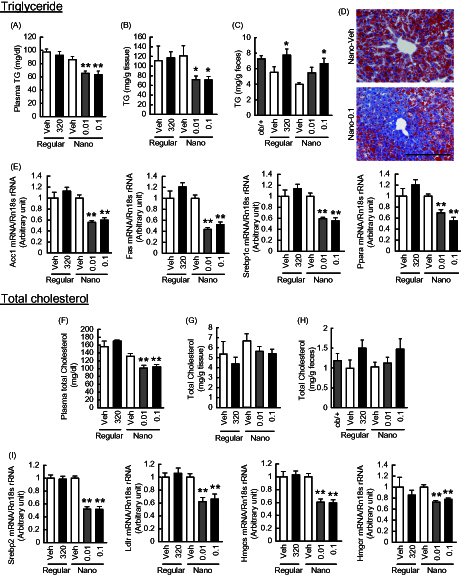
Effects of Nano-Orz on lipid metabolism in *ob*/*ob* mice. Metabolism of triglyceride (TG) (A–E) and total cholesterol (F–I) in Nano-Orz-treated *ob*/*ob* mice. Plasma (A, F), hepatic accumulation (B, G) and fecal (C, H) levels of TG (A–C) and total cholesterol (F–H). (D) Oil red O staining of liver sections. Scale bar, 200 μm; magnification, ×200. (E) Expression levels of lipogenic genes, ACC1, FAS, SREBP1c and PPARα in liver (*n* = 8). (I) Expression levels of genes involved in cholesterol synthesis, SREPB2, LDLR, HMGcs and HMGcr in liver (*n* = 8). The mRNA levels were determined by real-time PCR and normalized by those of 18S rRNA. Data are expressed as mean ± SEM. *p < 0.05, **p < 0.01 versus vehicle-treated mice (Veh). ANOVA followed by multiple comparison tests (Bonferroni/Dunn method) was used.

ER stress-induced insulin resistance in liver exaggerates lipogenesis and causes lipid dysmetabolism (Hotamisligil, 2010). To assess the lipid metabolism in mice treated with Nano-Orz, we measured triglyceride and total-cholesterol contents in liver, feces and plasma. Feces were collected during a week before sacrifice. Although regular γ-oryzanol showed no effect on plasma triglyceride and total-cholesterol levels, Nano-Orz significantly decreased plasma triglyceride and total-cholesterol levels (Figures 5A and F). In parallel, Nano-Orz significantly decreased hepatic triglyceride and total-cholesterol levels (Figures 5B and G). This observation was endorsed by oil red O staining of liver sections (Figure 5D). On the other hand, there were no changes in fecal triglyceride and total-cholesterol contents in mice treated with Nano-Orz (Figures 5C and H). These data suggest that Nano-Orz decreases lipid synthesis in liver, but exerts no apparent effect on lipid absorption. Therefore, we examined the expression levels of genes related to *de novo* fatty acid synthesis including acetyl-CoA carboxylase (ACC), fatty acid synthase (FAS), sterol receptor element-binding protein-1c (SREBP-1c), and peroxisome proliferator-activated receptor-α (PPARα) as well as cholesterol synthesis including sterol receptor element-binding protein-2 (SREBP2), low density lipoprotein receptor (LDLR), 3-hydroxy-3-methylglutaryl (HMG)-CoA synthase and HMG-CoA reductase. As expected, regular γ-oryzanol showed no effect on the expression levels of these genes (Figures 5E and I). On the other hand, Nano-Orz significantly decreased the mRNA levels of genes related to fatty acid and cholesterol synthesis in liver (Figures 5E and I).

To better understand the entire effect of Nano-Orz on liver, gene expression profile was evaluated in the liver from *ob*/*ob* mice treated with Nano-Orz. Data were quantified with the distribution free weighted method (DFW), and calculated from a comparison among the groups by the rank product method (Breitling et al., 2004). According to their false discovery rate (FDR) values (> 0.05), 82 probe sets that were up-regulated and 100 probe sets that were down-regulated in the regular γ-oryzanol-treated group relative to the vehicle-treated group were identified. On the other hand, 134 probe sets that were up-regulated and 81 probe sets that were down-regulated in the Nano-Orz treated group relative to the vehicle-treated group were identified. Of note, only 19 probe sets that were up-regulated and eight probe sets that were down-regulated were overlapped between regular γ-oryzanol-treated group and Nano-Orz-treated group.

To identify gene ontology (GO) terms, a gene-annotation enrichment analysis was performed using the online software program DAVID (the Database for Annotation, Visualization, and Integrated Discovery). The GO term significantly enriched in the genes regulated by regular γ-oryzanol included lipid metabolic process (GO: 0006629) and monocarboxylic acid metabolic process (GO: 002787) (Supplemental Table 2A), while the GO term significantly enriched in the genes regulated by Nano-Orz-treated group was lipid metabolic process (GO: 0006629) (Supplemental Table 2B). These data indicate that γ-oryzanol *per se* mainly ameliorates lipid dysmetabolism in liver and Nano-Orz further strengthens its metabolically beneficial effects, but raise a possibility that Nano-Orz may have additional impact on lipid metabolism via distinct mechanisms related to encapsulation in PLGA nanoparticles.

### Effects of Nano-Orz on gut microbiota in mice

To explore the possibility that Nano-Orz would exert its metabolically beneficial effects, at least partly, via the change in gut microbiota, we assessed the composition of gut microbiota in mice treated with Nano-Orz. There was no change in diversity of gut microbiota between *ob*/*+ *and *ob*/*ob* mice (Supplemental Figure 2A). Both regular γ-oryzanol and Nano-Orz showed no effect on diversity of gut microbiota (Supplemental Figure 2A). As previously shown (Turnbaugh et al., 2006), *Firmicutes*/*Bacteroidetes* ratio, which is increased in prevalent forms of obesity in humans, was apparently increased in *ob*/*ob* mice (Supplemental Figures 2B and 2C). Although regular γ-oryzanol showed no effect on *Firmicutes*/*Bacteroidetes* ratio, Nano-Orz dose dependently decreased the ratio (Supplemental Figures 2B and 2C).

Next, we measured plasma SCFA concentrations including acetic acid, propionic acid, n-butyric acid and n-valeric acid in mice treated with Nano-Orz. In *ob/ob* mice, there was no change in the plasma concentrations of acetic acid and n-butyric acid, while those of propionic acid and n-valeric acid were significantly decreased (Supplemental Figure 2D). Regular γ-oryzanol showed no effect on a line of SCFA concentrations in plasma (Supplemental Figure 2D). On the other hand, Nano-Orz showed a trend to increase plasma levels of propionic acid and n-valeric acid (Supplemental Figure 2D).

### Effects of Nano-Orz on the preference for dietary fat in mice

**Figure 6.**
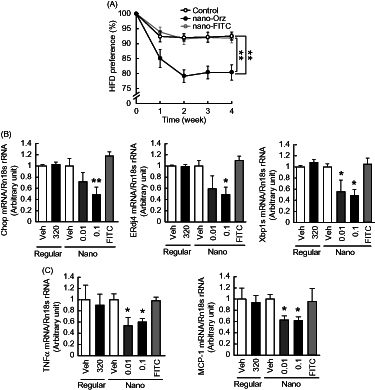
Impact of Nano-Orz on preference for dietary fat and hypothalamic ER stress in *ob*/*ob* mice. (A) HFD preference in mice treated with Nano-Orz. Mice were allowed free access to CD and HFD (*n* = 4; three mice per cage). Mice were treated with Nano-Orz (0.01 and 0.1 μg/g body weight/day) or FITC-encapsulated PLGA nanoparticles (0.1 μg/g body weight/day) for 4 weeks, and mRNA levels were measured in the hypothalamus for Chop, ERdj4, Xbp1s (B), TNFα and MCP-1 (C). Values were normalized to that of 18S rRNA and are expressed as levels relative to that of vehicle-treated mice (*n* = 8). The mRNA levels were determined using real-time PCR. *p < 0.05, **p < 0.01 compared with vehicle-treated mice. ANOVA followed by multiple comparison tests (Bonferroni/Dunn method) was used.

To investigate the effect of Nano-Orz on feeding behavior, we measured food consumption when mice were allowed to choose freely between the chow and HFD. There was no difference in total food intake between vehicle-treated mice and mice treated with Nano-Orz for 4 weeks. The vehicle-treated mice strongly preferred the HFD (Figure 6A). On the other hand, in mice treated with Nano-Orz (0.1 mg/kg body weight/day), HFD preference was decreased significantly (Figure 6A).

We previously showed that elevated hypothalamic ER stress does cause change in the preference for dietary fat in mice (Kozuka et al., 2012). We therefore examined the effect of Nano-Orz on hypothalamic ER stress. The mRNA levels of ER stress-responsive genes were significantly decreased in the hypothalamus by administration of Nano-Orz (Figure 6B). The mRNA levels of pro-inflammatory genes were also decreased in the hypothalamus in mice treated with Nano-Orz (Figure 6C). IHC was performed to visualize hypothalamic microglia, one of critical players of brain inflammation in response to dietary fats by use of specific antibodies against Iba-1. As previously reported (Gao et al., 2014), microglia was increased in hypothalamus of *ob*/*ob* mice by HFD (Supplemental Figure 3A and 3B). In contrast, this increment showed a trend to decrease by Nano-Orz (Supplemental Figures 3A and 3B).

Next, we evaluated gene expression profile in hypothalamus from *ob*/*ob* mice treated with Nano-Orz. According to their FDR values (> 0.05), 77 probe sets that were up-regulated and 173 probe sets that were down-regulated in the regular γ-oryzanol-treated group relative to the vehicle-treated group were identified. On the other hand, 109 probe sets that were up-regulated and 336 probe sets that were down-regulated in mice treated with Nano-Orz relative to the vehicle-treated group were identified. Of note, only five probe sets that were up-regulated and 22 that were down-regulated were overlapped between regular γ-oryzanol-treated group and Nano-Orz-treated group. The GO terms significantly enriched in the genes regulated by regular γ-oryzanol and Nano-Orz are shown in Supplemental Tables 3A and 3B. Canonical pathway analyses were performed using QIAGEN’s Ingenuity Pathway Analysis (IPA®, QIAGEN Redwood City, www.qiagen.com/ingenuity). Pathways with significantly enriched in respective genes (−log (*p* value) > 2.0 each) were extracted. The canonical pathway significantly enriched in the genes regulated by regular γ-oryzanol included 36 of signaling pathways among 59 pathways (Supplemental Table 4A). Since many of these pathways consisted of common molecules, these data indicate that effects of regular γ-oryzanol are focused on specific signaling pathways. In contrast, the canonical pathway significantly enriched in the genes regulated by Nano-Orz *per se* included only eight pathways (Supplemental Table 4B). These data indicate that effects of Nano-Orz influence nonspecifically and widely on various pathways. Some genes were regulated oppositely between regular γ-oryzanol and Nano-Orz, suggesting that Nano-Orz functions on hypothalamus via distinct mechanisms related to encapsulation in PLGA nanoparticles.

## Discussion

In this study, we performed in-depth evaluation of Nano-Orz toward obesity-diabetes syndrome in experimental mice models. Although γ-oryzanol has a wide variety of beneficially metabolic effects (Kozuka et al., 2012; Kozuka et al., 2013; Kozuka et al., 2015a, Kozuka et al., 2015b), extremely low absorption efficiency from intestine of γ-oryzanol is a tough obstacle for the clinical application. Gamma-oryzanol ameliorates preference for animal fat and fuel metabolism in HFD-fed mice (Kozuka et al., 2012, 2013, 2015a, 2015b). However, in *ob*/*ob* mice, the best-known severest diabetic model in mice, γ-oryzanol actually shows no apparent effect on metabolic amelioration. To fully bring out the potential effects of γ-oryzanol, we encapsulated γ-oryzanol in PLGA nanoparticle (Nano-Orz). To our surprise, Nano-Orz markedly ameliorated fuel metabolism with an unexpected magnitude (∼1000-fold lower dose) compared with regular γ-oryzanol in *ob*/*ob* mice (Figures 2–6).

PLGA is approved by the US Food and Drug Administration (FDA) and European Medicine Agency (EMA) for drug delivery use (Danhier et al., 2012). Previous *in vivo* lactate release studies showed that plasma level of lactate was increased four-fold by encapsulation with PLGA (Chereddy et al., 2016). In the tissue distribution study in Balb/C mice, after 7 days of oral administration of PLGA, nanoparticles remained detectable in the brain, heart, kidney, liver, lungs and spleen (Semete et al., 2010). On the other hand, in F344 rats, after 21 days of oral administration, the highest amounts of PLGA nanoparticles were found and showed minimal toxicity in the intestine, liver, kidney, lung and brain (Navarro et al., 2016). Of note, a previous study using C57BL/6 mice treated with ICG or Nano-ICG intravenously, ICG distributed mainly in the liver, followed by the kidneys, spleen, heart and lungs. On the other hand, nanoparticle formulation showed a distinct and longer bio-distribution pattern from that of Nano-ICG (Saxena et al., 2006). These studies suggest that PLGA can be exploited for sustained supply of composition.

In this study, we demonstrated that after 3 days of oral administration, Nano-ICG mainly distributed in the liver and intestine in C57BL/6J mice (Figure 1). It is likely that changes in molecular weight, particle size, total surface area and hydrophilic/lipophilic balance of PLGA may affect the rate of degradation and the tissue distribution (Trabold et al., 2003). According to previous studies showing that smaller particles are taken up to a greater extent than larger ones (McClean et al., 1998; Sahana et al., 2008), the particle size used in this study (approximately 200 nm) are anticipated to be absorbed with considerably high efficiency. Presumably reflecting such a benefit, Nano-Orz markedly ameliorated glucose and lipid metabolism in *ob*/*ob* mice (∼1000-fold efficiency) compared with regular γ-oryzanol.

Recent studies have shown that modulation of gut microbiota by unrefined whole grains results in metabolically beneficial changes in plasma levels of SCFA through bacterial fermentation (Rosenbaum et al., 2015). Noticeably, in most cases of human and rodent obesities, diversity of gut microbiota is decreased, and the ratio of *Firmicutes* to *Bacteroidetes* is elevated (Ley et al., 2005; Walters et al., 2014). A recent gnotobiotic study has shown that gut microbiota from leptin-deficient genetically obese mice (*Lep^ob^*) represents high proportion of *Firmicutes*, which correlates well with increase in energy harvest and decrease in energy expenditure, resulting in robust body fat gain in recipient mice (Turnbaugh et al., 2006). However, importantly, dietary habits primarily impact the variation of gut microbiota rather than genetic obesities *per se* (Murphy et al., 2010; Moreno-Indias et al., 2014). Consistent with a previous report (Murphy et al., 2010), in this study, *Firmicutes*/*Bacteroidetes* ratio was significantly increased in *ob*/*ob* mice compared with *ob*/+ mice (Supplemental Figure 2). On the other hand, expression of genes encoding enzymes involved in the initial breakdown step for indigestible dietary polysaccharides or enzymes which import and metabolize a line of end-products was significantly increased in the *ob*/*ob* microbiome (Turnbaugh et al., 2006; Moreno-Indias et al., 2014). All these data suggest the importance of the *Firmicutes*/*Bacteroidetes* ratio in the development of obesity in both humans and rodents. In this study, Nano-Orz did decrease the *Firmicutes*/*Bacteroidetes* ratio (Supplemental Figure 2B). Moreover, plasma SCFA level showed a trend toward increase in Nano-Orz-treated mice (Supplemental Figure 2D). SCFAs produced by microbiota-driven fermentation are transported into the intestinal lumen and affect glucose and lipid metabolism in various tissues (den Besten et al., 2013). In this study, Nano-Orz effectively decreased ER stress and inflammation also in liver and adipose tissue (Figures 4 and 5), and ameliorated glucose, triglyceride and cholesterol metabolism in *ob*/*ob* mice (Figures 3, 4 and 5). Microarray data in liver further reinforced the impact of Nano-Orz on lipid metabolism. In addition to the marked increase in the absorption efficiency of Nano-Orz, these findings raise a possibility that Nano-Orz ameliorates obesity and glucose dysmetabolism at least partly via the considerable changes in gut microbiota and subsequent production of SCFAs. In this context, further studies are warranted to test this hypothesis.

From a viewpoint of clinical applications, it is important to note that Nano-Orz significantly decreased ER stress in hypothalamus and attenuated the preference for dietary fat by administration once every 2 weeks (Figure 6). According to a previous study on bio-distribution of PLGA nanoparticles in mice (Semete et al., 2010), the particles remained detectable in the brain after 7 days of oral administration. Furthermore, we have shown that the full structure of γ-oryzanol plays a crucial role in reduction of ER stress and consequent attenuation of the preference for dietary fat in mice (Kozuka et al., 2012). Taken together, it is reasonable to speculate that Nano-Orz reaches the brain and regulates feeding behavior in hypothalamus. Microarray data in hypothalamus showing the wide differences in the GO term between regular γ-oryzanol and Nano-Orz may also support this hypothesis, but precise molecular mechanisms must await further investigation.

In summary, we demonstrate that Nano-Orz markedly ameliorate glucose and lipid metabolism in obese diabetic *ob*/*ob* mice with an unexpected magnitude. Furthermore, such conspicuous impacts on fuel dyshomeostasis are achievable by its administration once every 2 weeks. Nanotechnology-based developments of functional foods with health claims or drug discoveries oriented toward γ-oryzanol shed light on the novel approach for the prevention and treatment of lifestyle-related metabolic diseases in humans.

## Supplementary Material

161230_Nano_Suppl_Drug_Delivery.docx

## Notes

**Supplementary material available online**
