# Supplementary material for: Marked augmentation of PLGA nanoparticle-induced metabolically beneficial impact of γ-oryzanol on fuel dyshomeostasis in genetically obese-diabetic ob/ob mice
Source: Drug Deliv. 2017 Feb 9;24(1):558–68. doi: 10.1080/10717544.2017.1279237 (PMC8241037; doi:10.1080/10717544.2017.1279237)
Supplement: 161230_Nano_Suppl_Drug_Delivery.docx [file IDRD_A_1279237_SM9475.docx]

Marked augmentation of PLGA nanoparticle-induced metabolically-beneficial impact of γ-oryzanol on fuel dyshomeostasis in genetically obese-diabetic *ob*/*ob* mice

Chisayo Kozuka^1^, Chigusa Shimizu-Okabe^2^, Chitoshi Takayama^2^, Kaku Nakano^3, 4^, Hidetaka Morinaga^5^, Ayano Kinjo^5^, Kotaro Fukuda^5^, Asuka Kamei^6^, Akihito Yasuoka^6^, Takashi Kondo^6^, Keiko Abe^6, 7^, Kensuke Egashira^3, 4, 5^ and Hiroaki Masuzaki^1^

**Supplemental Material**

**Supplemental Methods**

***Animals***

Whole blood was taken from the tail vein and blood glucose was measured using an　automatic glucometer (Medisafe Mini; Terumo, Tokyo, Japan). Occasional blood　sampling were done from the retro-orbital venous plexuses or tail vein. Plasma insulin　levels were measured using ELISA kits (Morinaga Institute of Biological Science, Inc.,　Tokyo, Japan). For OGTTs, mice were orally administrated with 0.75 g/kg body weight　glucose after an 18-h fast. Blood glucose levels were measured at the indicated times.

***PLGA nanoparticles***

PLGA and each compound were dissolved in acetone/ethanol solution completely. The　polymer solution was dropped into stirring aqueous PVA solution. The organic solvent　in the resultant mixture was removed by a rotary evaporator. Finally, the evaporated　mixture was filtered and powdered by freeze drying methods. PLGA with an average　molecular weight of 20,000 and a lactide to glycolide copolymer ratio of 75:25　(PLGA7520; Wako Pure Chemical Industries, Osaka, Japan) was used as a wall　material for nanoparticle formation.

***IHC analyses and oil red O staining***

The dissected pancreas was fixed in 4 % paraformaldehyde, embedded in paraffin and sectioned. The paraffin-embedded sections were stained with hematoxylin and eosin (H&E) or immunostained for insulin (ab7842, Abcam Japan, Tokyo, Japan), glucagon (G2654, Sigma-Aldrich, St Louis, MO, USA), and cleaved caspase-3 (9661; Cell Signaling Technology Japan, Tokyo, Japan). The mean size and ratio of inulin-positive and glucagon-positive areas to the total islet area were calculated based on >100 islets per group using Photoshop (Adobe, San Jose, CA, USA). For ionized calcium binding adaptor protein-1 (Iba-1) IHC staining, frozen brain sections were stained with anti-Iba-1 (019-19741; Wako Pure Chemical Industries). Six immunohistochemically stained sections in each group were captured using the Scanscope XT image scanner (Aperio Technologies, Vista, CA, USA) and analyzed using the ImageScope program.

***Quantitative real-time PCR***

Gene expression was examined as described (Kozuka *et al.*, 2012). Total RNA was extracted using Trizol reagent (Life technologies Japan, Tokyo, Japan) and cDNA was synthesized using an iScript™ cDNA Synthesis Kit (Bio-Rad, Hercules, CA, USA). Quantitative real-time PCR was performed using a StepOnePlusTM Real-Time PCR System, and Fast SYBR Green Master Mix (Life technologies). The mRNA levels were normalized by *Rn18s* (18S rRNA). The primer sets used for the quantitative real-time PCR analyses were summarized in **Supplemental Table 1**.

***MicroArray Analyses***

For assays, 3 representative mice were selected in each group. DNA microarray analysis was performed as previously described (Kamei *et al.*, 2013). Briefly, total RNA from liver and hypothalamus was assessed for quality and quantity by agarose gel electrophoresis and ultraviolet spectrophotometry, respectively. DNA microarray analysis was performed according to the manufacturer’s instructions (Affymetrix, Santa Clara, CA, USA). The fluorescent signal intensities were measured with an Affymetrix GeneChip® Scanner 3000 7G. The Affymetrix® GeneChip® Command Console® (AGCC) software program (Affymetrix, Santa Clara, CA, USA) was used to convert the array images into intensity values for each probe (CEL files).

**Supplemental References:**

Kamei, A., Watanabe, Y., Kondo, K., Okada, S., Shinozaki, F., Ishijima, T., Nakai, Y., Kondo, T., Arai, S. & Abe, K., 2013. Influence of a short-term iron-deficient diet on hepatic gene expression profiles in rats. *PLoS One,* 8**,** e65732.

Kozuka, C., Yabiku, K., Sunagawa, S., Ueda, R., Taira, S.I., Ohshiro, H., Ikema, T., Yamakawa, K., Higa, M., Tanaka, H., Takayama, C., Matsushita, M., Oyadomari, S., Shimabukuro, M. & Masuzaki, H., 2012. Brown Rice and Its Component, gamma-Oryzanol, Attenuate the Preference for High-Fat Diet by Decreasing Hypothalamic Endoplasmic Reticulum Stress in Mice. *Diabetes,* 61**,** 3084-3093.

**Supplemental Tables**

**Supplemental Table 1. The primer sets used for quantitative real-time PCR analysis**

| Gene | Primer (5′ to 3′) |
| --- | --- |
| *Drd2* | *f* CCA TTG TCT GGG TCC TGT CC |
| (D2R) | *r* GTG GGT ACA GTT GCC CTT GA |
| *Slc6a3* | *f* GCA CTA CTT CTT CTC CTC CT |
| (DAT) | *r* CCT GAA GTC TTT ACT CCC TTC C |
| *Th* | *f* CCC TAC CAA GAT CAA ACC TAC C |
| (TH) | *r* GAG CGC ATG CAG TAG TAA GA |
| *Slc18a2* | *f* GTC TGT CTA TGG GAG TGT GTA T |
| (VMAT2) | *r* GGG TAC GGC TGG ACA TTA TT |
| *Ddit3* | *f* CCA CCA CAC CTG AAA GCA GAA |
| (Chop) | *r* AGG TGA AAG GCA GGG ACT CA |
| *Dnajb9* | *f* CCC CAG TGT CAA ACT GTA CCA G |
| (ERdj4) | *r* AGC GTT TCC AAT TTT CCA TAA ATT |
| *Xbp1s* | *f* AGTTAAGAACACGCTTGGGAATGG |
|  | *r* CTGCTGCAGAGGTGCACATAGTC |
| *Ppargc1a* | *f* TAT GGA GTG ACA TAG AGT GTG CT |
| (PCG1α) | *r* CCA CTT CAA TCC ACC CAG AAA G |
| *Pck* | *f* CTG CAT AAC GGT CTG GAC TTC |
| (PEPCK) | *r* CAG CAA CTG CCC GTA CTC C |
| *G6pc* | *f* CGA CTC GCT ATC TCC AAG TGA |
| (G6Pase) | *r* GTT GAA CCA GTC TCC GAC CA |
| *Tnf* | *f* TCT CTT CAA GGG ACA AGG CTG |
| (TNFα) | *r* ATA GCA AAT CGG CTG ACG GT |
|  | *p* CCC GAC TAC GTG CTC CTC ACC CA |
| *Il6* | *f* ATG AAG TTC CTC TCT GCA AGA G |
| (IL-6) | *r* GTA GGG AAG GCC GTG GTT G |
|  | *p* CAC CAG CAT CAG TCC CAA GAA GGC A |
| *Ccl2* | *f* TTG GCT CAG CCA GAT GCA |
| (MCP-1) | *r* CCA GCC TAC TCA TTG GGA TCA |
|  | *p* CCC CAC TCA CCT GCT GCT ACT CAT TCA |
| *Pparg* | *f* TGG GTG AAA CTC TGG GAG ATT C |
| (PPARγ2) | *r* AAT TTC TTG TGA AGT GCT CAT AGG C |
|  | *p* CCT GTT GAC CCA GAG CAT GGT GCC |
| *Acaca* | *f* GCC ATT GGT ATT GGG GCT TAC |
| (ACC1) | *r* CCC GAC CAA GGA CTT TGT TG |
|  | *p* GCT GGG ACA AAG AAC CAT CCA GGT TGA |
| *Fasn* | *f* GGC TCA GCA TGG TCG CTT |
| (FAS) | *r* CTC CCG CCA GCT GTC ATT |
|  | *p* AAC CAC CCT CTG GGC ATG GCT ATC TTC T |
| *Srebf1* | *f* GGA CAC AGC GGT TTT GAA CG |
| (SREBP1c) | *r* CCT GTC TCA CCC CCA GCA TA |
|  | *p* CAG CTC ATC AAC AAC CAA GAC AGT GAC TTC |
| *Ppara* | *f* CTG TTT GTG GCT GCT ATA ATT TGC |
| (PPARα) | *r* CCT GCA ACT TCT CAA TGT AGC C |
|  | *p* TGG AGA TCG GCC TGG CCT TCT AAA CAT |
| *Srebf2* | *f* CAC AAT ATC ATT GAA AAG CGC TAC CGG TCC |
| (SREBP2) | *r* TTT TTC TGA TTG GCC AGC TTC AGC ACC ATG |
| *Ldlr* | *f* GAA GTC GAC ACT GTA CTG ACC ACC |
| (LDL-R) | *r* CTC CTC ATT CCC TCT GCC AGC CAT |
| *Hmgcs* | *f* AAC TGG TGC AGA AAT CTC TAG C |
| (HMG CoA synthase 1) | *r* GGT TGA ATA GCT CAG AAC TAG CC |
| *Hmgcr* | *f* AGC TTG CCC GAA TTG TAT GTG |
| (HMG CoA reductase) | *r* TCT GTT GTG AAC CAT GTG ACT TC |
| *Rn18s* | *f* TTC TGG CCA ACG GTC TAG ACA AC |
| (18S rRNA) | *r* CCA GTG GTC TTG GTG TGC TGA |

Forward and reverse primers are designated by *f* and *r*, respectively.

Probe (FAM-5' -> 3'-TAMRA) is designated by *p*.

**Supplemental Table 2. GO terms that were significantly enriched (FDR-corrected P < 0.01) in the liver transcriptome data.**

|  | GO ID | GO term | | | | | | | | | FDR-corrected *P*-value |
| --- | --- | --- | --- | --- | --- | --- | --- | --- | --- | --- | --- |
| (A) Significantly enriched GO terms (FDR-corrected *P*-value < 0.01) found in 82 probe sets that were up-regulated and 100 probe sets that were down-regulated in the regular γ-oryzanol-treated group relative to the vehicle-treated group. | | | | | | | | | | | |
|  | GO:0008150 | biological process | | | | | | | | - | |
|  | GO:0008152 |  | metabolic process | | | | | | | - | |
|  | GO:0044238 |  |  | primary metabolic process | | | | | | - | |
|  | GO:0071704 |  |  | organic substance metabolic process | | | | | | - | |
|  | GO:0006629 |  |  |  | lipid metabolic process | | | | | 0.001 | |
|  | GO:0044699 |  | single-organism process | | | | | | | - | |
|  | GO:0009987 |  | cellular process | | | | | | | - | |
|  | GO:0044710 |  |  | single-organism metabolic process | | | | | | - | |
|  | GO:0044281 |  |  |  | small molecule metabolic process | | | | | - | |
|  | GO:0044237 |  |  |  | cellular metabolic process | | | | | - | |
|  | GO:0044763 |  |  |  | single-organism cellular process | | | | | - | |
|  | GO:0006082 |  |  |  |  | organic acid metabolic process | | | | 0.109 | |
|  | GO:0043436 |  |  |  |  |  | oxoacid metabolic process | | | 0.114 | |
|  | GO:0019752 |  |  |  |  |  |  | carboxylic acid metabolic process | | 0.114 | |
|  | GO:0032787 |  |  |  |  |  |  |  | monocarboxylic acid metabolic process | 0.009 | |
|  |  |  |  |  |  |  |  |  |  |  | |
| (B) Significantly enriched GO terms (FDR-corrected *P*-value < 0.01) found in 134 probe sets that were up-regulated and 81 probe sets that were down-regulated in the Nano-Orz treated group relative to the vehicle-treated group. | | | | | | | | | | | |
|  | GO:0008150 | biological process | | | | | | | | - | |
|  | GO:0008152 |  | metabolic process | | | | | | | - | |
|  | GO:0044699 |  | single-organism process | | | | | | | - | |
|  | GO:0071704 |  |  | organic substance metabolic process | | | | | | - | |
|  | GO:0044238 |  |  | primary metabolic process | | | | | | - | |
|  | GO:0044710 |  |  | single-organism metabolic process | | | | | | - | |
|  | GO:0006629 |  |  |  | lipid metabolic process | | | | | 8.78E-04 | |

GO terms with no *P*-value indicate no significance.

FDR-corrected *P*-values of the categories exhibiting the deepest hierarchy are shadowed.

**Supplemental Table 3. GO terms that were significantly enriched (FDR-corrected P < 0.01) in the hypothalamus transcriptome data.**

|  | GO ID | GO term | | | | | | | | | | FDR-corrected *P*-value |
| --- | --- | --- | --- | --- | --- | --- | --- | --- | --- | --- | --- | --- |
| (A) Significantly enriched GO terms (FDR-corrected *P*-value < 0.01) found in 77 probe sets that were up-regulated and 173 probe sets that were down-regulated in the regular γ-oryzanol-treated group relative to the vehicle-treated group. | | | | | | | | | | | | |
|  | GO:0008150 | biological process | | | | | | | | | | - |
|  | GO:003250 |  | developmental process | | | | | | | | | 0.007 |
|  | GO:0048856 |  |  | anatomical structure development | | | | | | | | 0.003 |
|  | GO:0044767 |  |  | single-organism developmental process | | | | | | | | - |
|  | GO:0044707 |  |  | single-multicellular organism process | | | | | | | | - |
|  | GO:0007275 |  |  |  | multicellular organismal development | | | | | | | 0.004 |
|  | GO:0048731 |  |  |  |  | system development | | | | | | 0.001 |
|  | GO:0007399 |  |  |  |  |  | nervous system development | | | | | 0.004 |
|  | GO:0048513 |  |  |  |  |  | organ development | | | | | 0.005 |
|  | GO:0009887 |  |  |  |  |  |  | organ morphogenesis | | | | 0.004 |
|  | GO:003250 |  | multicellular organismal process | | | | | | | | | 0.003 |
|  | GO:0044699 |  | single-organism process | | | | | | | | | - |
|  | GO:0044763 |  |  | single-organism cellular process | | | | | | | | - |
|  | GO:0048869 |  |  |  | cellular developmental process | | | | | | | 0.243 |
|  | GO:0030154 |  |  |  |  | cell differentiation | | | | | | 0.199 |
|  | GO:0048468 |  |  |  |  |  | cell development | | | | | 0.005 |
|  | GO:0050896 |  | response to stimulus | | | | | | | | | 0.058 |
|  | GO:0042221 |  |  | response to chemical stimulus | | | | | | | | 0.195 |
|  | GO:0010033 |  |  |  | response to organic substance | | | | | | | 0.004 |
|  | GO:0065007 |  | biological regulation | | | | | | | | | 0.000 |
|  | GO:0009987 |  | cellular process | | | | | | | | | - |
|  | GO:0050789 |  |  | regulation of biological process | | | | | | | | 0.002 |
|  | GO:0050794 |  |  |  | regulation of cellular process | | | | | | | 0.006 |
|  | GO:0048518 |  |  |  | positive regulation of biological process | | | | | | | 0.001 |
|  | GO:0044237 |  |  |  | cellular metabolic process | | | | | | | - |
|  | GO:0048522 |  |  |  |  | positive regulation of cellular process | | | | | | 0.003 |
|  | GO:0031323 |  |  |  |  | regulation of cellular metabolic process | | | | | | 0.065 |
|  | GO:0031325 |  |  |  |  |  | positive regulation of cellular metabolic process | | | | | 0.010 |
|  | GO:0071704 |  |  | organic substance metabolic process | | | | | | | | - |
|  | GO:0019222 |  |  |  | regulation of metabolic process | | | | | | | 0.066 |
|  | GO:0043170 |  |  |  | macromolecule metabolic process | | | | | | | - |
|  | GO:0009653 |  |  |  |  | anatomical structure morphogenesis | | | | | | 0.022 |
|  | GO:0009893 |  |  |  |  | positive regulation of metabolic process | | | | | | 0.003 |
|  | GO:0060255 |  |  |  |  | regulation of macromolecule metabolic process | | | | | | 0.112 |
|  | GO:0010604 |  |  |  |  |  | positive regulation of macromolecule metabolic process | | | | | 0.002 |
|  |  |  |  |  |  |  |  |  |  |  |  |  |
| (B) Significantly enriched GO terms (FDR-corrected *P*-value < 0.01) found in 109 probe sets that were up-regulated and 336 probe sets that were down-regulated in mice treated with Nano-Orz relative to the vehicle-treated group. | | | | | | | | | | | | |
|  | GO:0008150 | biological process | | | | | | | | | | - |
|  | GO:0032502 |  | developmental process | | | | | | | | | 0.008 |
|  | GO:0044699 |  | single-organism process | | | | | | | | | - |
|  | GO:0032501 |  | multicellular organismal process | | | | | | | | | 0.001 |
|  | GO:0009987 |  | cellular process | | | | | | | | | - |
|  | GO:0044767 |  |  | single-organism developmental process | | | | | | | | - |
|  | GO:0044707 |  |  | single-multicellular organism process | | | | | | | | - |
|  | GO:0044763 |  |  | single-organism cellular process | | | | | | | | - |
|  | GO:0048869 |  |  |  | cellular developmental process | | | | | | | 0.134 |
|  | GO:0030154 |  |  |  |  | cell differentiation | | | | | | 0.151 |
|  | GO:0009888 |  |  |  |  | tissue development | | | | | | 0.153 |
|  | GO:0048468 |  |  |  |  |  | cell development | | | | | 0.029 |
|  | GO:0048863 |  |  |  |  |  | stem cell differentiation | | | | | - |
|  | GO:0060485 |  |  |  |  |  |  | mesenchyme development | | | | 0.032 |
|  | GO:0048864 |  |  |  |  |  |  |  | stem cell development | | | - |
|  | GO:0048762 |  |  |  |  |  |  |  | mesenchymal cell differentiation | | | 0.030 |
|  | GO:0014032 |  |  |  |  |  |  |  |  | neural crest cell development | | 0.008 |
|  | GO:0014031 |  |  |  |  |  |  |  |  | mesenchymal cell development | | 0.027 |
|  | GO:0014033 |  |  |  |  |  |  |  |  |  | neural crest cell differentiation | 0.008 |
|  | GO:0048856 |  |  | anatomical structure development | | | | | | | | 0.000 |
|  | GO:0001503 |  |  |  | ossification | | | | | | | 0.009 |
|  | GO:0007275 |  |  |  | multicellular organismal development | | | | | | | 0.007 |
|  | GO:0035295 |  |  |  |  | tube development | | | | | | 0.008 |
|  | GO:0048731 |  |  |  |  | system development | | | | | | 0.001 |
|  | GO:0009653 |  |  |  |  | anatomical structure morphogenesis | | | | | | 0.007 |
|  | GO:0048513 |  |  |  |  |  | organ development | | | | | 0.001 |
|  | GO:0001501 |  |  |  |  |  | skeletal system development | | | | | 0.008 |
|  | GO:0007399 |  |  |  |  |  | nervous system development | | | | | 0.009 |
|  | GO:0009887 |  |  |  |  |  |  | organ morphogenesis | | | | 0.009 |
|  | GO:0060348 |  |  |  |  |  |  | bone development | | | | 0.006 |
|  | GO:0065007 |  | biological regulation | | | | | | | | | 0.001 |
|  | GO:0050789 |  |  | regulation of biological process | | | | | | | | 0.018 |
|  | GO:0051239 |  |  |  | regulation of multicellular organismal process | | | | | | | 0.007 |
|  | GO:0048519 |  |  |  | negative regulation of biological process | | | | | | | 0.007 |
|  | GO:0048518 |  |  |  | positive regulation of biological process | | | | | | | 0.005 |
|  | GO:0050896 |  | response to stimulus | | | | | | | | | 0.004 |
|  | GO:0042221 |  |  | response to chemical stimulus | | | | | | | | 0.060 |
|  | GO:0010033 |  |  |  | response to organic substance | | | | | | | 0.008 |

GO terms with no P-value indicate no significance.

FDR-corrected P-values of the categories exhibiting the deepest hierarchy are shadowed.

**Supplemental Table 4. IPA canonical pathways enriched (-log(p-value) > 2.0) in the hypothalamus transcriptome data.**

|  | Ingenuity Canonical Pathways | -log  (p-value) | Ratio | z-score | Molecules |
| --- | --- | --- | --- | --- | --- |
| (A) Significantly enriched canonical pathways found in 77 probe sets that were up-regulated and 173 probe sets that were down-regulated in the regular γ-oryzanol-treated group relative to the vehicle-treated group. | | | | | |
|  | Aldosterone Signaling in Epithelial Cells | 5.20E+00 | 6.85E-02 | -2.646 | PLCB4,SGK1,DNAJC27,PRKCD,SLC12A2,SOS1,DNAJC18,PLCB1,PRKCH,HSPA4L |
|  | Melatonin Signaling | 4.08E+00 | 9.38E-02 | -2.236 | BRAF,PLCB4,RORA,PRKCD,PLCB1,PRKCH |
|  | Cholecystokinin/Gastrin-mediated Signaling | 3.94E+00 | 7.22E-02 | -2.646 | FOS,PLCB4,PRKCD,SOS1,PLCB1,PRKCH,CCK |
|  | Prolactin Signaling | 3.82E+00 | 8.45E-02 | -2.000 | FOS,PRKCD,SOS1,CREBBP,PRKCH,NR3C1 |
|  | VEGF Family Ligand-Receptor Interactions | 3.76E+00 | 8.22E-02 | -0.816 | FOS,FLT1,PRKCD,SOS1,FIGF,PRKCH |
|  | Molecular Mechanisms of Cancer | 3.73E+00 | 3.69E-02 | NaN | FZD3,CREBBP,BMPR2,HIF1A,APC,BRAF,FOS,PLCB4,PRKCD,SOS1,PLCB1,PRKCH,GNAL |
|  | GNRH Signaling | 3.35E+00 | 5.79E-02 | -2.646 | FOS,PLCB4,PRKCD,SOS1,CREBBP,PLCB1,PRKCH |
|  | Factors Promoting Cardiogenesis in Vertebrates | 3.34E+00 | 6.90E-02 | NaN | FZD3,PRKCD,BMPR2,PRKCH,APC,TCF7L2 |
|  | Endothelin-1 Signaling | 3.31E+00 | 4.97E-02 | -2.828 | BRAF,FOS,PLCB4,PRKCD,SOS1,PLCB1,PRKCH,GNAL |
|  | Production of Nitric Oxide and Reactive Oxygen Species in Macrophages | 3.24E+00 | 4.85E-02 | -2.121 | FOS,ALB,PRKCD,APOA2,PPM1L,CREBBP,SERPINA1,PRKCH |
|  | Mouse Embryonic Stem Cell Pluripotency | 3.19E+00 | 6.45E-02 | -1.633 | FZD3,SOS1,CREBBP,BMPR2,APC,TCF7L2 |
|  | Neuropathic Pain Signaling In Dorsal Horn Neurons | 3.14E+00 | 6.32E-02 | -2.449 | FOS,PLCB4,NTRK2,PRKCD,PLCB1,PRKCH |
|  | Pyridoxal 5'-phosphate Salvage Pathway | 3.14E+00 | 7.94E-02 | NaN | BRAF,FAM20B,SGK1,PRKCD,PRKCH |
|  | Synaptic Long Term Depression | 3.12E+00 | 5.30E-02 | -1.134 | PLCB4,PRKCD,PPM1L,CRH,PLCB1,PRKCH,GNAL |
|  | Huntington's Disease Signaling | 3.08E+00 | 4.15E-02 | NaN | PLCB4,SGK1,PRKCD,SOS1,CREBBP,PLCB1,TCERG1,PRKCH,RPH3A |
|  | Corticotropin Releasing Hormone Signaling | 2.93E+00 | 5.77E-02 | -1.633 | BRAF,FOS,PRKCD,CREBBP,CRH,PRKCH |
|  | BMP signaling pathway | 2.90E+00 | 7.04E-02 | -2.000 | SOSTDC1,SOS1,CREBBP,BMPR2,PITX2 |
|  | UVC-Induced MAPK Signaling | 2.90E+00 | 9.52E-02 | -2.000 | BRAF,FOS,PRKCD,PRKCH |
|  | Role of Macrophages, Fibroblasts and Endothelial Cells in Rheumatoid Arthritis | 2.90E+00 | 3.60E-02 | NaN | FOS,PLCB4,FZD3,PRKCD,CREBBP,PLCB1,FIGF,PRKCH,APC,TCF7L2 |
|  | GPCR-Mediated Nutrient Sensing in Enteroendocrine Cells | 2.77E+00 | 6.58E-02 | NaN | PLCB4,PRKCD,PLCB1,PRKCH,CCK |
|  | FXR/RXR Activation | 2.77E+00 | 5.36E-02 | NaN | TTR,ALB,APOA2,CREBBP,SERPINA1,GC |
|  | IL-12 Signaling and Production in Macrophages | 2.77E+00 | 5.36E-02 | NaN | FOS,ALB,PRKCD,APOA2,SERPINA1,PRKCH |
|  | Bupropion Degradation | 2.73E+00 | 1.36E-01 | NaN | CYP2E1,CYP3A5,CYP2C8 |
|  | P2Y Purigenic Receptor Signaling Pathway | 2.71E+00 | 5.22E-02 | -2.449 | FOS,PLCB4,PRKCD,CREBBP,PLCB1,PRKCH |
|  | p70S6K Signaling | 2.71E+00 | 5.22E-02 | -1.633 | PLCB4,PRKCD,PPM1L,SOS1,PLCB1,PRKCH |
|  | Acute Phase Response Signaling | 2.68E+00 | 4.46E-02 | -0.447 | FOS,TTR,ALB,APOA2,SOS1,SERPINA1,NR3C1 |
|  | Acetone Degradation I (to Methylglyoxal) | 2.67E+00 | 1.30E-01 | NaN | CYP2E1,CYP3A5,CYP2C8 |
|  | Wnt/β-catenin Signaling | 2.61E+00 | 4.32E-02 | -0.816 | SOX17,FZD3,PPM1L,CREBBP,BMPR2,APC,TCF7L2 |
|  | CREB Signaling in Neurons | 2.58E+00 | 4.27E-02 | -2.236 | PLCB4,PRKCD,SOS1,CREBBP,PLCB1,PRKCH,GNAL |
|  | TGF-β Signaling | 2.56E+00 | 5.88E-02 | -1.000 | FOS,SOS1,CREBBP,BMPR2,PITX2 |
|  | PPARα/RXRα Activation | 2.56E+00 | 4.24E-02 | 1.134 | PLCB4,APOA2,SOS1,CREBBP,BMPR2,PLCB1,CYP2C8 |
|  | Estrogen Receptor Signaling | 2.56E+00 | 4.88E-02 | NaN | TAF1,THRAP3,SOS1,CREBBP,NR3C1,PGR |
|  | Salvage Pathways of Pyrimidine Ribonucleotides | 2.56E+00 | 5.88E-02 | NaN | BRAF,FAM20B,SGK1,PRKCD,PRKCH |
|  | VEGF Signaling | 2.54E+00 | 5.81E-02 | 0.447 | ACTA2,FLT1,SOS1,FIGF,HIF1A |
|  | Thrombopoietin Signaling | 2.49E+00 | 7.41E-02 | -2.000 | FOS,PRKCD,SOS1,PRKCH |
|  | Ovarian Cancer Signaling | 2.49E+00 | 4.72E-02 | NaN | BRAF,FZD3,FIGF,CGA,APC,TCF7L2 |
|  | Wnt/Ca+ pathway | 2.46E+00 | 7.27E-02 | -2.000 | PLCB4,FZD3,CREBBP,PLCB1 |
|  | mTOR Signaling | 2.44E+00 | 4.02E-02 | -1.000 | PRKCD,PPM1L,Rn18s,FIGF,PRKCH,HIF1A,EIF4G1 |
|  | Human Embryonic Stem Cell Pluripotency | 2.44E+00 | 4.62E-02 | NaN | NTRK2,ZIC3,FZD3,BMPR2,APC,TCF7L2 |
|  | Breast Cancer Regulation by Stathmin1 | 2.33E+00 | 3.85E-02 | NaN | PLCB4,PRKCD,PPM1L,SOS1,RB1CC1,PLCB1,PRKCH |
|  | Glioblastoma Multiforme Signaling | 2.26E+00 | 4.23E-02 | -1.633 | PLCB4,FZD3,PRKCD,SOS1,PLCB1,APC |
|  | Erythropoietin Signaling | 2.20E+00 | 6.15E-02 | NaN | FOS,PRKCD,SOS1,PRKCH |
|  | Renal Cell Carcinoma Signaling | 2.20E+00 | 6.15E-02 | NaN | FOS,SOS1,CREBBP,HIF1A |
|  | Neurotrophin/TRK Signaling | 2.20E+00 | 6.15E-02 | NaN | FOS,NTRK2,SOS1,CREBBP |
|  | GPCR-Mediated Integration of Enteroendocrine Signaling Exemplified by an L Cell | 2.20E+00 | 6.15E-02 | NaN | PLCB4,PLCB1,CCK,VIP |
|  | Gap Junction Signaling | 2.20E+00 | 4.11E-02 | NaN | PLCB4,ACTA2,PRKCD,SOS1,PLCB1,PRKCH |
|  | Tec Kinase Signaling | 2.18E+00 | 4.08E-02 | -1.342 | FOS,ACTA2,PRKCD,VAV3,PRKCH,GNAL |
|  | Estrogen Biosynthesis | 2.18E+00 | 8.82E-02 | NaN | CYP2E1,CYP3A5,CYP2C8 |
|  | CXCR4 Signaling | 2.17E+00 | 4.05E-02 | -2.000 | FOS,PLCB4,PRKCD,PLCB1,PRKCH,GNAL |
|  | LXR/RXR Activation | 2.13E+00 | 4.67E-02 | -2.236 | TTR,ALB,APOA2,SERPINA1,GC |
|  | IL-3 Signaling | 2.11E+00 | 5.80E-02 | NaN | FOS,PRKCD,SOS1,PRKCH |
|  | Dopamine-DARPP32 Feedback in cAMP Signaling | 2.10E+00 | 3.92E-02 | -2.236 | PLCB4,PRKCD,PPM1L,CREBBP,PLCB1,PRKCH |
|  | Sperm Motility | 2.10E+00 | 4.59E-02 | -2.000 | PLCB4,PRKCD,SLC12A2,PLCB1,PRKCH |
|  | Transcriptional Regulatory Network in Embryonic Stem Cells | 2.05E+00 | 7.89E-02 | NaN | GBX2,ZIC3,SIX3 |
|  | 14-3-3-mediated Signaling | 2.04E+00 | 4.42E-02 | -2.236 | FOS,PLCB4,PRKCD,PLCB1,PRKCH |
|  | Synaptic Long Term Potentiation | 2.02E+00 | 4.39E-02 | -2.236 | PLCB4,PRKCD,CREBBP,PLCB1,PRKCH |
|  | Mechanisms of Viral Exit from Host Cells | 2.02E+00 | 7.69E-02 | NaN | ACTA2,PRKCD,PRKCH |
|  | Thyroid Cancer Signaling | 2.02E+00 | 7.69E-02 | NaN | BRAF,NTRK2,TCF7L2 |
|  | PTEN Signaling | 2.01E+00 | 4.35E-02 | 0.447 | NTRK2,FLT1,SOS1,BMPR2,FOXG1 |
|  |  |  |  |  |  |
| (B) Significantly enriched canonical pathways found in 109 probe sets that were up-regulated and 336 probe sets that were down-regulated in mice treated with Nano-Orz relative to the vehicle-treated group. | | | | | |
|  | Hepatic Fibrosis / Hepatic Stellate Cell Activation | 4.50E+00 | 7.56E-02 | NaN | COL1A2,COL1A1,IGFBP4,COL8A2,IGF2,CTGF,FN1,COL6A2,LEPR,COL9A3,COL8A1,LBP,COL3A1 |
|  | Acute Phase Response Signaling | 4.24E+00 | 7.64E-02 | 0.000 | FOS,SERPING1,TTR,FN1,NFKBIA,ITIH2,CP,LBP,CRABP2,RBP1,RBP4,SERPIND1 |
|  | Intrinsic Prothrombin Activation Pathway | 3.82E+00 | 1.85E-01 | -2.000 | COL1A2,COL1A1,F5,THBD,COL3A1 |
|  | Extrinsic Prothrombin Activation Pathway | 2.47E+00 | 1.88E-01 | NaN | F5,TFPI,THBD |
|  | Coagulation System | 2.34E+00 | 1.14E-01 | 0.000 | F5,TFPI,THBD,SERPIND1 |
|  | Atherosclerosis Signaling | 2.23E+00 | 6.31E-02 | NaN | COL1A2,COL1A1,LYZ,CXCL12,COL3A1,RBP4,APOD |
|  | Dendritic Cell Maturation | 2.11E+00 | 5.48E-02 | -1.890 | COL1A2,COL1A1,NFKBIA,LEPR,HLA-DQA1,LY75,HLA-DRB5,COL3A1 |
|  | IGF-1 Signaling | 2.02E+00 | 6.45E-02 | NaN | IGFBP4,FOS,IGFBP6,CTGF,NOV,IGFBP2 |

**Supplemental Figures**

**Supplemental Figure 1.**

**Effects of Nano-Orz on gene expressions related to inflammation and ER stress in mesenteric fat from *ob*/*ob* mice**

(**A-C**) Expression levels of mRNA for TNFα, IL-6, MCP-1 (**A**), Chop, ERdj4, Xbp1s (**B**), and PPARγ2 (**C**) in mesenteric fat (*n* = 8). The mRNA levels were determined by real-time PCR and normalized by those of *Rn18s*. Data are expressed as mean ± SEM. * *P* < 0.05, ** *P* < 0.01, *vs* vehicle-treated mice (Veh).

**Supplemental Figure 2.**

**Effects of Nano-Orz on gut microbiota and plasma SCFA level**

Diversity of cecal gut microbiota was analyzed using 16S rRNA sequencing in *ob*/*ob* mice treated with Nano-Orz for 4 weeks. OTU numbers (**A**), bars represent the relative abundance (%) of bacterial phyla (**B**) and the *Firmicutes*/*Bacteroidetes* ratio (**C**) are shown for each group. (**D**) Plasma SCFA levels in Nano-Orz-treated *ob*/*ob* mice. Data are expressed as mean ± SEM.

**Supplemental Figure 3.**

**Effects of Nano-Orz on hypothalamic Iba-1 positive microglia**

(A) Iba-1 staining of hypothalamus in brain sections. Scale bar, 1 mm; magnification, x40. (B) Iba-1-positive area was calculated in 6 sections in each group. Data are expressed as mean ± SEM.
